# Using Six Sigma DMAIC Methodology and Discrete Event Simulation to Reduce Patient Discharge Time in King Hussein Cancer Center

**DOI:** 10.1155/2018/3832151

**Published:** 2018-06-24

**Authors:** Mazen Arafeh, Mahmoud A. Barghash, Nirmin Haddad, Nadeem Musharbash, Dana Nashawati, Adnan Al-Bashir, Fatina Assaf

**Affiliations:** ^1^The Department of Industrial Engineering, The University of Jordan, Amman, Jordan; ^2^King Hussein Cancer Center, Amman, Jordan; ^3^The Department of Industrial Engineering, The Hashemite University, Zarqa, Jordan

## Abstract

Short discharge time from hospitals increases both bed availability and patients' and families' satisfaction. In this study, the Six Sigma process improvement methodology was applied to reduce patients' discharge time in a cancer treatment hospital. Data on the duration of all activities, from the physician signing the discharge form to the patient leaving the treatment room, were collected through patient shadowing. These data were analyzed using detailed process maps and cause-and-effect diagrams. Fragmented and unstandardized processes and procedures and a lack of communication among the stakeholders were among the leading causes of long discharge times. Categorizing patients by their needs enabled better design of the discharge processes. Discrete event simulation was utilized as a decision support tool to test the effect of the improvements under different scenarios. Simplified and standardized processes, improved communications, and system-wide management are among the proposed improvements, which reduced patient discharge time by 54% from 216 minutes. Cultivating the necessary ownership through stakeholder analysis is an essential ingredient of sustainable improvement efforts.

## 1. Introduction

Overcrowding in emergency departments (ED) is a problem in many countries around the world [1]. ED overcrowding has been reported to cause delays in diagnosis, delays in treatment, decreased quality of care, and poor patient outcomes [2, 3]. The main causes of ED overcrowding seem to originate outside the ED [4]; patients are held in the emergency department after they have been admitted to the hospital because no inpatient beds are available [5]. The lack of inpatient beds is the most significant reason for ED overcrowding [5, 6]. One strategy hospitals have used to combat overcrowding is investing in new construction and additional staffing. Another strategy, which offers the potential for greater return on investment, is process improvement [7].

We chose to use the latter approach in tackling the patient discharge process. A lengthy, inefficient process for discharging patients is a common concern for hospitals. Good discharge management is vital to ensure patient satisfaction, as well as bed availability for emergency and elective admissions [8].

6*σ* is both a quality management philosophy and a methodology that focuses on reducing variation, measuring defects, and improving the quality of products, processes, and services [9]. The foundations of 6*σ* were established by Bill Smith at Motorola Corporation in response to product quality challenges in the late 1980s [10]. It was further developed by General Electric in the late 1990s [11]. 6*σ* projects are formalized and highly structured, making use of scientific approaches in the selection and management of projects. 6*σ* projects use a Define-Measure-Analyze-Improve-Control (DMAIC) structure, considered by many practitioners to be the primary reason for 6*σ*'s success [12].

Although Six Sigma (6*σ*) originated in manufacturing in late 1980s [13], it has been successfully applied in the healthcare field in an effort to improve processes and remedy inefficiencies. The literature has several examples that tackle numerous problems, including reducing medical errors [14], improving pharmacist dispensing errors [15], lessening medication dispensing time [16], identifying variables affecting the risk of healthcare associated infections and decreasing the percentage of patients with healthcare associated infections [17, 18], and decreasing the length of stay and treatment imaging [19] just to mention few.

Because the discharge process is stochastic in nature, simulation provides a vibrant platform to capture the dynamic and complex features and to predict the consequences of potential improvement efforts. This happens to be the entirety of the project through the steps designed to understand the system, build a model, run the model, and analyze the results using appropriate statistics. Discrete event simulation (DES), a computerized method of imitating the operation of a real-world system over time, can provide decision makers with an evidence-based tool to develop and objectively vet operational solutions prior to implementation [20]. DES was developed in the 1960s in industrial engineering and operations research to help analyze and improve industrial and business processes [21]. A benefit of using DES is the ability to incorporate multiple performance measures associated with healthcare systems to help to understand the relationships between various inputs [22]. The use of simulation is growing and is seen as a powerful tool for the healthcare industry, able to model a wide range of topic areas and answer a variety of research questions [23–25]. DES in health care commonly focuses on (1) improving patient flow, (2) managing bed capacity, (3) scheduling staff, (4) managing patient admission and scheduling procedures, and (5) using ancillary resources (e.g., labs and pharmacies) [20].

In this paper, we present a holistic approach that combines DES and stakeholder analysis under the umbrella of the 6*σ* DMAIC framework to examine the discharge process of patients in a hospital.

The rest of the paper is organized as follows. The next section provides a review of the literature on patient discharge processes and stakeholders analysis. Section three presents a background on the project and a brief description of the methodology used. Section four discusses the application of the 6*σ* DMAIC methodology in detail including (an overview of the discharge process, the DES model, and the improvement scenarios and results). The final section provides concluding remarks.

## 2. Literature Review

Because of the complex nature of the discharge process, only a few authors have examined the application of 6*σ* to the improvement of the discharge process in hospitals. Allen et al. [26] described the application of 6*σ* to streamlining patient discharge at a community hospital in Alliance, Ohio, United States. Their findings suggested that focusing on physician preparation for writing discharge orders would have the greatest impact. Using several tools, including statistical process control charting, process mapping, Pareto charting, and cause-and-effect matrices to analyze and solve the problem, they reported a reduction in the average discharge time from 3.3 to 2.8 hours.

Although Niemeijer et al. [27] titled their work “Quality in trauma care: improving the discharge procedure of patients by means of Lean Six Sigma,” the work actually addressed the reduction of the average length of stay of patients at the University Medical Center Groningen in the northern part of the Netherlands. Here we do not undermine their efforts and findings, we only point out that their work was not specifically aimed directly at improving the discharge process.

Udayai and Kumar [8] attempted to reduce the discharge time of cash patients at an Indian hospital based on results from analysis of voice of the customers (patients). The authors conducted a time-motion study to measure each step in the process to determine factors impacting the overall process. Improvements included starting billing one hour earlier and assigning priority for patients “pending discharge” on the computer that listed patients who needed an X-ray, lab report, or medicines.

El-Banna [28] presented a case study in which he built a simulation model of the discharge process at a private hospital in Amman, Jordan. He focused on insured patients in all three departments of the hospital (female, male, and pediatrics). He then optimized the model with a designed experiment and response surface model. He found that insurance and pharmacy operations were critical in the process. The author reported that the patient discharge time was decreased to be less than 50 minutes, which increased customer satisfaction, increased the number of admissions and turnovers on the rooms, and increased the hospital's profitability.

Vijay [29] suggested various improvement strategies to reduce the cycle time of patients' discharge process in a multidisciplinary hospital setting in India using the 6*σ* Define, Measure, Analyze, Improve, and Control (DMAIC) model. The study identified five critical issues causing delays in the timely handover of the discharge summary to the patients. The issues were failure to utilize information technology to generate and verify the patient information, job rotation, lack of decentralized discharge summary preparation process, failure to empower the assistant physician or surgeon to proof read the rough discharge note prepared by the editor for review, and failure to link all the computers located in all the departments with ERP software so that accurate and up-to-date information about the patients can be gathered without delay. These issues were further explored and subjected to root cause analysis using brainstorming techniques. A 61% reduction in the cycle time of the discharge process was then achieved by removing non-value-added activities.

Rossi et al. [30] utilized the Lean 6*σ* methodology to improve discharge room cleaning processes. Large delays in turnover of patient rooms and inconsistent cleaning practices were occurring because of a lack of knowledge about cleaning roles and responsibilities and because of a lack of communication between the services.

Table 1 summarizes the articles that have addressed the discharge process, highlighting the different tools used.

Notably, in the case studies presented in these articles, only one used simulation as a tool and none included stakeholder analysis. This work attempts to present a comprehensive approach that includes tools that can detect problems and failures, tools that measure current and future performance and tools that help generate new solutions. This work also uses stakeholder analysis.

Stakeholders are critical to the success of Lean Six Sigma (LSS) projects [31, 32]. Regardless of its technical justification, any change effort needs sufficient support and involvement from key stakeholders. The ability to mobilize commitment often makes the difference between a success and a good idea that failed [33].

Stakeholder analysis identifies the stakeholder groups, their roles, how they are impacted, and their concerns related to the process [9]. A stakeholder is anyone impacted by the project; however, the project sponsor and project manager need to identify the key stakeholders needed to support, promote, and sustain the project and its improvement.

Stakeholder analysis enhances the ownership of the project's success (including sustaining the improvement) among the stakeholders and improves communication [34]. The main aim in performing a stakeholder analysis is to understand the stakeholders' attitudes toward change and potential reasons for resistance. The next step is to develop activities, plans, and actions that can help the team to overcome resistance and barriers to change. It is used to help ensure that the entire organization will accept and be comfortable with the improvement initiative and the changes that it is proposing. All stakeholders are analyzed to try and identify any issues or concerns that they may have with the new improvement strategies. You can then develop a strategy to address these potential barriers so that the targeted processes and areas can be changed effectively.

## 3. Project Background and Methodology Overview

This 6*σ* project was implemented at King Hussein Cancer Center (KHCC), a 262-bed hospital specialized in cancer treatment in Amman, Jordan. This public nongovernmental hospital provides high-quality services to patients of more than 48 nationalities from around the world. The hospital is famous for its provision of diagnostic, therapeutic, and healthcare services to different types of cancer patients with different needs. In 2016, approximately 8,722 patients were discharged from the hospital. Delays in discharging patients affected the hospital operations and impacted the overcrowded ED throughput since many patients in the ED await to be admitted to the hospital.

6*σ* DMAIC is used in addition to DES to help to clarify the problems in the patient discharge process. The 6*σ* DMAIC approach was applied for process improvement in five phases: (1) the “Define” phase, where the objectives were defined and a project charter was made; (2) the “Measure” phase, where shadowing at the KHCC took place for real-time data observations through the eyes of patients and their families, resulting in enough information to draw a process flow map and a supplier-input-process-output-customer (SIPOC) diagram; (3) the “Analyze” phase, which utilized the fishbone diagram, the five whys, and the communication plan, as well as the implementation of the simulation model and validation using the ProModel software package to detect long-duration activities and to try to reduce them and to eliminate the non-value-added activities if they exist; (4) the “Improve” phase, the phase of change, where all possible improvements were made to minimize the total discharge duration; and (5) the “Control” phase, where the benefits of using the improvement model were described so that KHCC can take suitable actions regarding this issue.

## 4. Application of Six Sigma DMAIC Methodology

The project was managed by a 6*σ* Black Belt (BB) following the DMAIC roadmap. The BB ensured that each improvement tools were used appropriately during each phase. The BB also verified that the project's solutions were correct and complete.

### 4.1. “Define” Phase

The project BB and Champion described and scoped the project. They also met with selected team members, explaining the project objectives and importance, discussing their roles, and listening to their feedback. The team decided to focus on medical and surgical patients. Preliminary data analysis showed that medical patients had longer discharge times than did surgical patients, who have planned discharges, and for this reason, surgical patients were excluded from this study.

**Figure 1:**
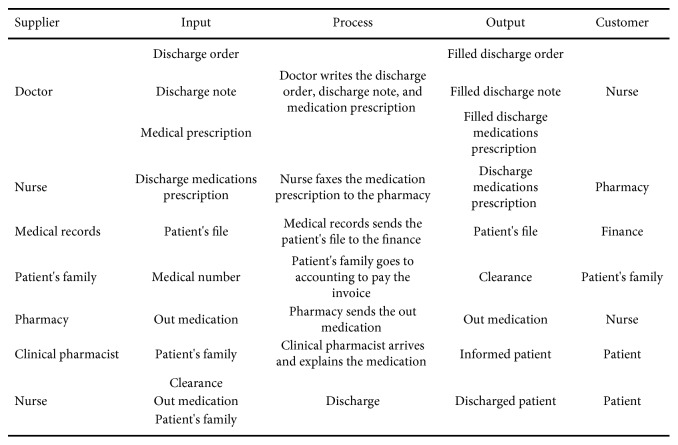
SIPOC diagram for the discharge process.

Process mapping was essential for understanding the process. A clear overview of the discharge process scope was provided with help of a SIPOC analysis, as shown in Figure 1. The SIPOC analysis included the macro process steps and identified all of the suppliers and customers involved in the process.

Developing the SIPOC analysis provided the team with an understanding of the project's major components and boundaries. The patient discharge process was defined as the set of activities that started with a specialist's signature on the discharge order and ended with a patient leaving the room. The team prepared a project charter, which was approved to proceed to the next step.

### 4.2. “Measure” Phase

The “Measure” phase began with preparing detailed process maps and data collection, followed by analyzing the initial state and conducting a process capability analysis of the discharge process. The key measure in this phase was the time of all the activities starting from the physician's signature on the discharge form and ending when the patient left the room.

To create a detailed process flowchart, team members shadowed patients and gathered real-time data observations through the eyes of patients and their families. Since we are dealing with cancer patients, most patients were accompanied by their family members during the discharge process. Patients and/or their families are henceforth referred to as “PF.” The process map enabled the team to understand the process and to pinpoint potential bottlenecks and areas of variation in the discharge process.

**Figure 2:**
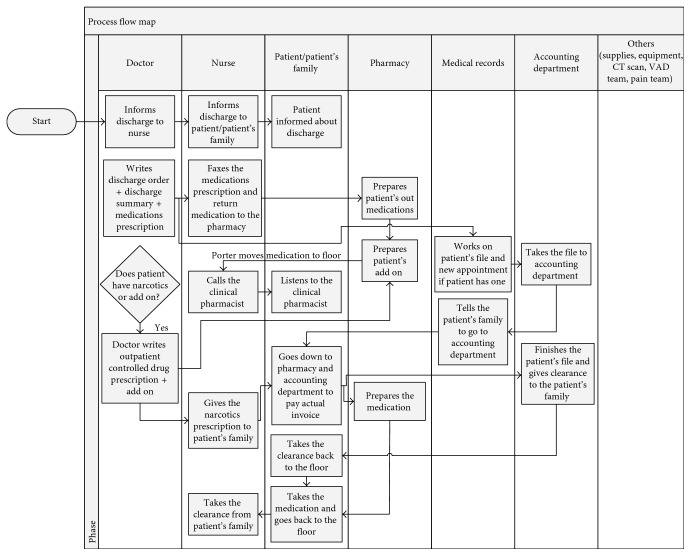
Discharge process flow map (part 1).

**Figure 3:**
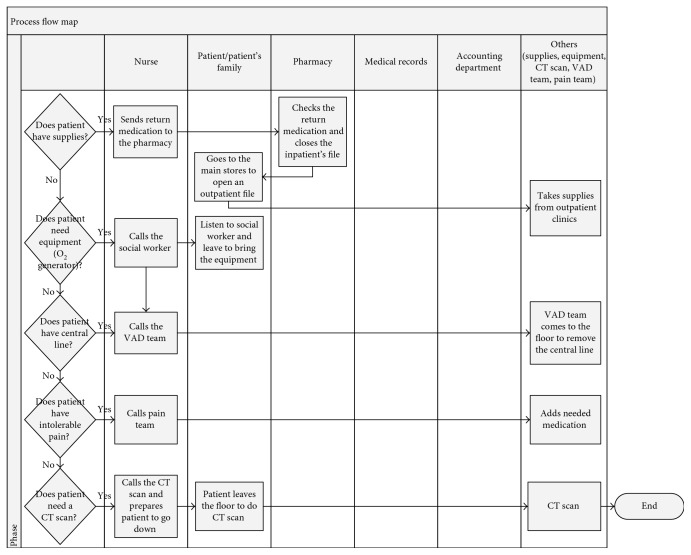
Discharge process flow map (part 2).

Figures 2 and 3 present a process flow map detailing the discharge process workflow. The figure presents one of the many scenarios that a discharged patient may experience. The process begins after the doctors finish their rounds and decide which patients are to be discharged. The nurse waits for the doctor to write the prescription for medication and then faxes it to the pharmacy, where the medication is prepared. After some time, the doctor writes the discharge order, which allows the medical records department to start working on the patient's file. A porter then takes the file to the accounting department on the ground floor, and the medical records department instructs the PF to pay the patient's bill and generates the clearance sheet indicating that the patient has no outstanding bills. After the medication is ready at the pharmacy and the porter has arrived, the medication is delivered to the nurses' station. The nurse contacts the clinical pharmacist, who provides counseling to the patient regarding the medication. If the doctor has forgotten a medication, an add-on prescription is written, and the process repeats. Before closing the inpatient file, extra medications that were prescribed to the patient but not used during the hospital stay must be returned to the pharmacy.

There are cases where the patient may have extra needs, such as the following:

- For narcotics, the physician also writes an outpatient controlled drug prescription, which the nurse delivers to the PF, who then submit it to the pharmacy. This step may occur early or late in the discharge process, depending on the physician.
- When supplies are issued after the inpatient file has been closed, the PF need to go to the outpatient clinic to create an outpatient file to buy the supplies.
- For the removal of a central IV line, the nurse contacts the venous access device team.
- When patients require equipment such as an oxygen generator, the nurse contacts a social worker, who suggests places where the family can buy the needed equipment.

Other needs such as sickness reports and settlement of billing questions are addressed in the Patient Affairs Office/Admissions, where the reports are printed. Some patients request sickness reports for medical leave purposes, these reports are then signed by the physician in charge.

The data were collected using approved and pretested data collection methods. Data on the discharge process were collected for a period of one month. The data collected included activities and durations. Observations regarding the activities were also noted. Of the discharges that took place, a sample of 41 patient discharges were closely shadowed. The selection was random from different floors. Each day 2 or 3 patients were shadowed. Three discharges were cancelled due to errors in measurements, leaving us with 38 patient discharges.

We calculated the mean and standard deviation of the sample, 215 minutes and 67 minutes, respectively. The minimum sample size needed was calculated using a confidence level of 95% and an error of 30 minutes (approximately half the standard deviation) using following equation [35]:

(1) $$\begin{array}{c} n={\left(\frac{1.96\times 67}{30}\right)}^{2}=20\text{ patients}. \end{array}$$

However, since we had 38 discharges, we decided to end the data collection and move forward to data analysis.

**Figure 4:**
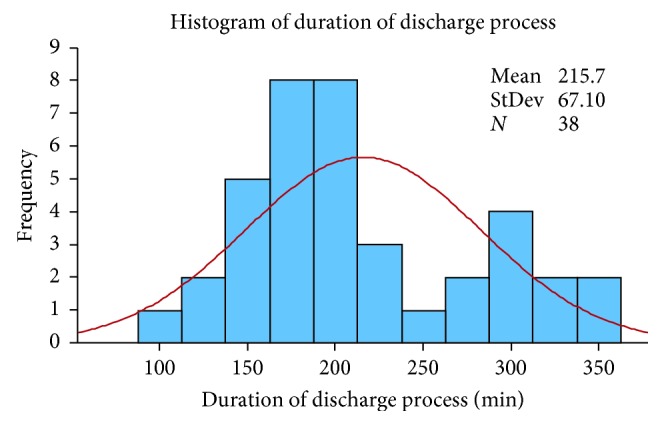
Histogram of the two populations.

Initial analysis of the data identified two populations, as shown in Figure 4.

The first population represents patients who go through the standard discharge process, which includes medication preparation, clinical pharmacist counseling, and accounting. The second population represents patients with extra needs (prescribed equipment, supplies, or add-on medications). We define discharges belonging to the first population as standard discharges and discharges belonging to the second population as complex discharges.

**Figure 5:**
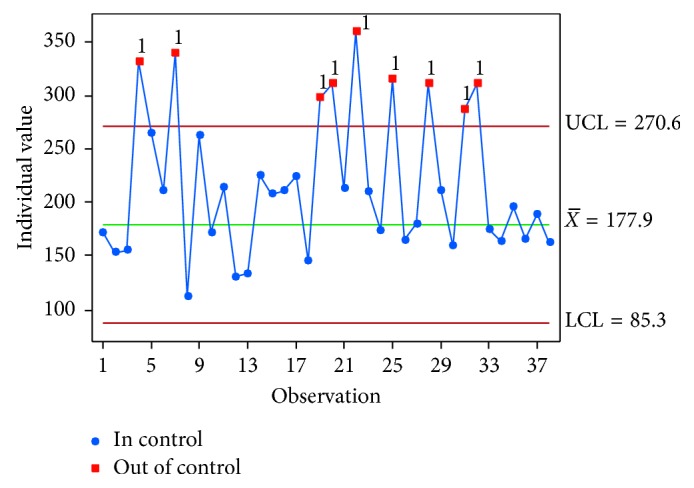
IMR control chart (collected data).

An individual control chart was drawn to identify the presence of special-cause variations in the discharge process, as shown in Figure 5. The typical discharge process takes about three hours. The out-of-control points (marked in red in the figure) are data points belonging to the second population.

**Figure 6:**
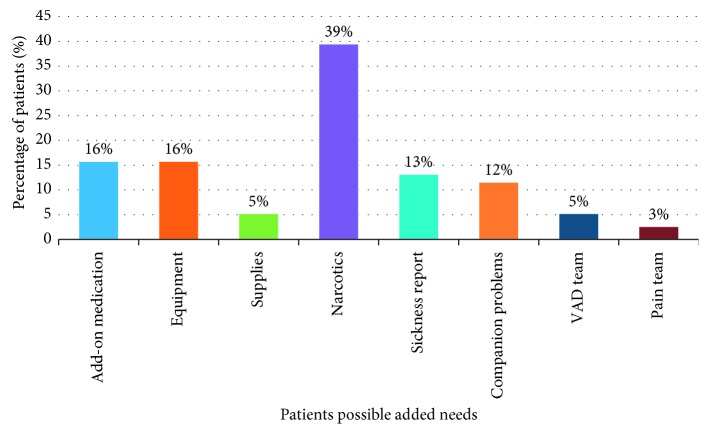
Classification of patients' needs (percentages).

To analyze observations of the process through the eyes of the patients, patients were classified according to their needs, as shown in Figure 6.

**Figure 7:**
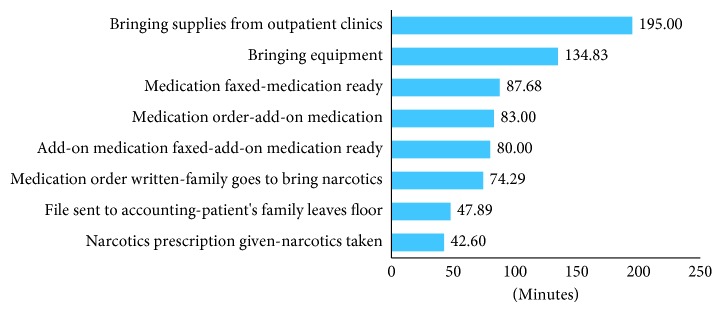
Duration of discharge process activities (minutes).

The average time spent on each activity was recorded, and a sample of these results is shown in Figure 7.

The following are some of the observations noted:

- Approximately 40% of patients needed narcotics (Figure 6), which adds an average of 43 minutes (Figure 7).
- Approximately 16% of patients needed special equipment, adding an average of 134 minutes.
- Approximately 5% of patients needed supplies from the outpatient clinic, requiring an average of 195 minutes.
- Additional medication was ordered for approximately 16% of patients; on average, this occurs 83 minutes after the first prescription is written.
- The duration between writing the discharge order and the medication order is about 38 minutes.

**Figure 8:**
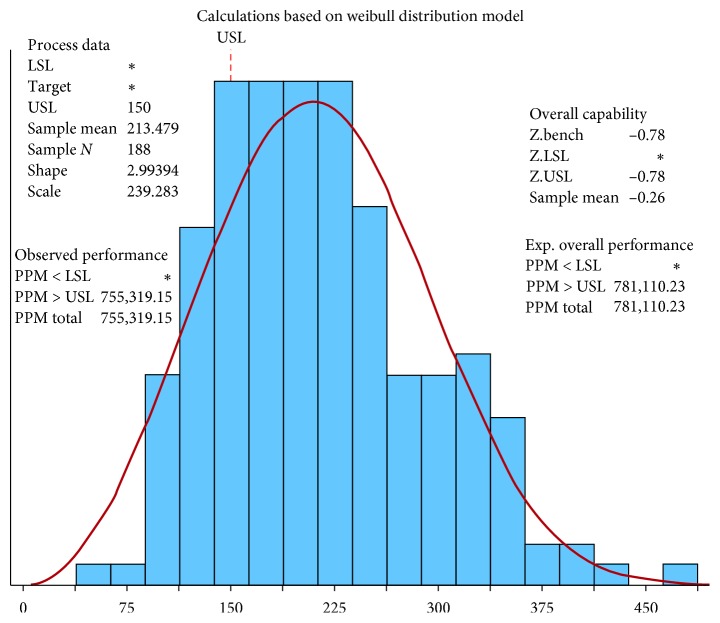
Process capability report before improvement.

A process capability analysis was performed to assess the performance of the discharge process. The main purpose of a capability study is to determine whether a process is capable of meeting certain requirements [36]. Capability analysis involves the calculation of the percentage of defects in the process and their corresponding sigma quality level (SQL).  Figure 8 shows the results of the process capability analysis performed using Minitab®; the resultant *Z*_bench_ of −0.78 is equivalent to an SQL of 0.72.

### 4.3. “Analyze” Phase

**Figure 9:**
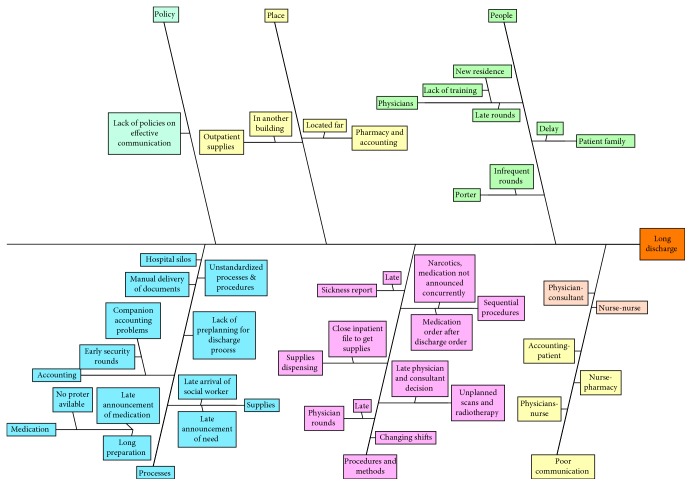
Cause-and-effect diagram.

After observing the discharge process and collecting data, efforts in the “Analyze” phase were focused on investigating the root causes of the problems in the processes. Observations noted while shadowing patients (data collection) followed by brainstorming sessions were used to examine potential reasons behind long discharge durations. A summary of the findings is presented using a cause-and-effect diagram (Figure 9). Some root causes identified in the figure actually were observed and some were identified as potential trying to holistically encompass the causes.

For example, in one rare case, the discharge process was delayed because of late arrival of the patient family.

Unnecessary variation in the discharge process and a lack of standardization led to two important undesirable outcomes: increased discharge time and decreased quality, where mistakes were likely to occur and people were likely to forget. Discharge orders, medication orders, and supplies and equipment orders were made in different sequences and at different times. Additionally, a prescription was sent to the pharmacy for preparation, but then later during the discharge process, another prescription order was sent to the pharmacy for the same patient for another medication, causing the discharge process to take longer than necessary.

Furthermore, a lack of preplanning for the discharge process and issues with the hospital layout were also among the root causes of the problem. A major cause of the long discharge process was poor communication between the different stakeholders (treating physicians, consultants, nurses, pharmacy staff, and the accounting department).

**Figure 10:**
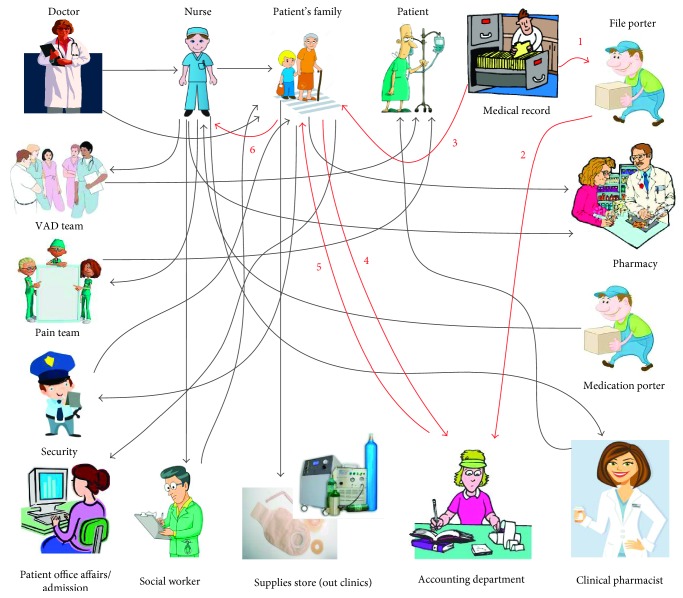
Communication complexity diagram.

Because the discharge process is a highly people-dependent process, it was imperative to observe the complexity of the communication in the discharge process. A communication complexity diagram is shown in Figure 10. For example, the medical records communicate with the porter, who in turn communicates with the accounting department. Communication between the accounting department back and forth with the PF and the PF with the nurse is also shown. These partial communications, highlighted in red and numbered 1–6, represent part of the communication cycle emphasizing the complex and multiplex details of the overall discharge process.

Observed delays caused by lapses in communication were noted in organizing the radiotherapy session, the inpatient chemotherapy regimen, the necessary diagnostic lab tests, and the diagnostic radiology imaging.

Delays caused by hospital security-PF-accounting department communications sometimes occurred because of PF challenging the fees added for companions. PF often denied having companions stay with the patient overnight, claiming that they had visitors who were incorrectly counted as companions when they visited after the last security rounds and refusing to pay the charges. In these cases, PF challenged the charges and requested a revision.

### 4.4. “Improve” Phase

In the “Improve” phase, the team examined the current state of process maps in depth, using brainstorming and cause-and-effect analysis techniques to explore possible solutions.

The improvement efforts included many facets. Figure 6 shows that about 20% of patients discharged from the hospital could be classified as complex discharges; they required special equipment or supplies at discharge. The remaining 80% of discharged patients were classified as standard discharges; they were discharged without the need for special equipment or supplies, and their discharge could be accomplished without complex planning. Changing how discharge occurs for both groups of patients will have a major impact on patient flow and the effective use of bed capacity. This can mean the difference between a system where patients experience long delays and one where delays are minimal.

The effective management of system-wide processes that support patient flow, such as admission, assessment and treatment, patient transfer, and discharge, can minimize delays in the delivery of care [37].

To support the improvement efforts and address the lack of standardization, checklists were proposed as a way to standardize the processes and ensure that all medications are prescribed together, thus eliminating the unnecessary delay caused by adding new medications to a patient's medication list. A thorough review of all medications should be an essential part of discharge planning. Effective discharge planning can ensure that medications are prescribed correctly.

According to “Discharge, Referral and Follow up” Standard no. 4.3, under “Access to Care and Continuity of Care,” planning for referral and/or discharge should begin early in the care process [37]. Diligent discharge planning has been associated with positive outcomes, including higher patient satisfaction [38].

However, despite the fact that it clearly increases the well-being of patients and caregivers, discharge planning is often not given the attention it deserves. Indeed, inefficient planning often adds to patients' and caregivers' stress. Effective discharge planning is crucial for ensuring timely discharge and making sure that the hospital's limited resources are used most effectively. Under the best of circumstances, the discharge planner should begin his or her evaluation when the patient is admitted to the hospital.

Furthermore, it is recognized that all departments involved in the discharge of a patient, from the pharmacy to the transport services, must collaborate to reduce overlap, waste, and frequent frustrations [39]. The role of discharge planning coordinator may be assigned to administrative staff, rotating-shift nurses, or full-time coordinator nurses. Three main roles are assigned to the coordinator: communication, multidisciplinary teamwork, and assessment. The inclusion of such a coordinator leads to successful process improvement efforts in non-physician-centered processes without interrupting physician care [40].

#### 4.4.1. Discrete Event Simulation

**Figure 11:**
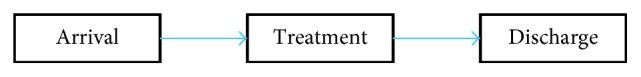
High-level process flowchart.

We tested several solutions to the problems identified in the “Analyze” phase using DES. The discharge process was modeled using ProModel 6.0 software. To build a complete simulation model, the simulation starts with the patient's arrival at the hospital for treatment and progresses through the receipt of treatment. Then, the discharge process is initiated, as shown in Figure 11.

**Figure 12:**
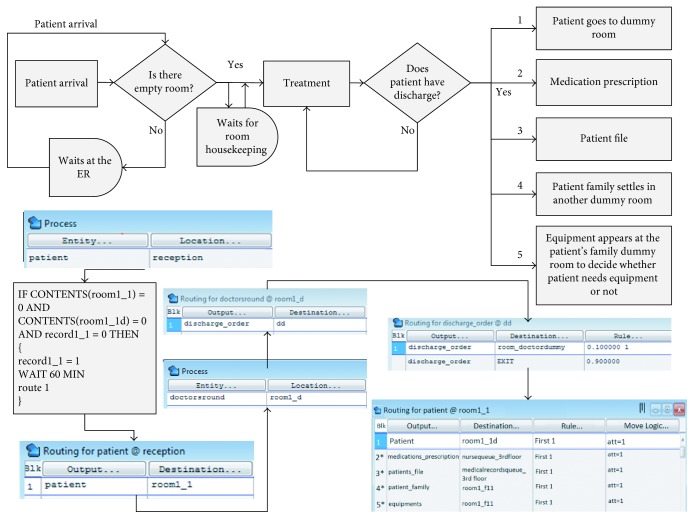
Patient discharge in the simulation model.

**Figure 13:**
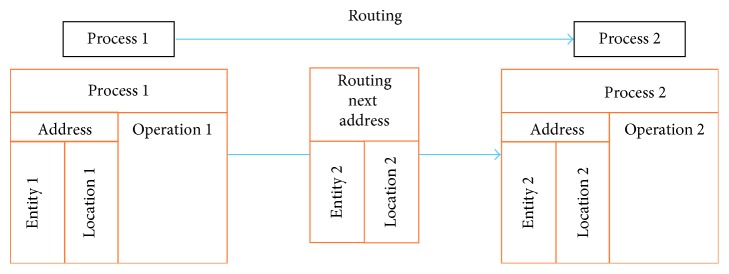
ProModel process definition.

We focus here on the discharge process.  Figure 12 presents a detailed process flowchart describing the simulation model from patient arrival to discharge. In ProModel, a *process* is initially defined by an *entity* and a *location* at which the *operation* is performed as shown in Figure 13. The operation defines the procedure performed in the process and the *routing*, which defines the *outcome entity* of the operation and where it is sent.

**Figure 14:**
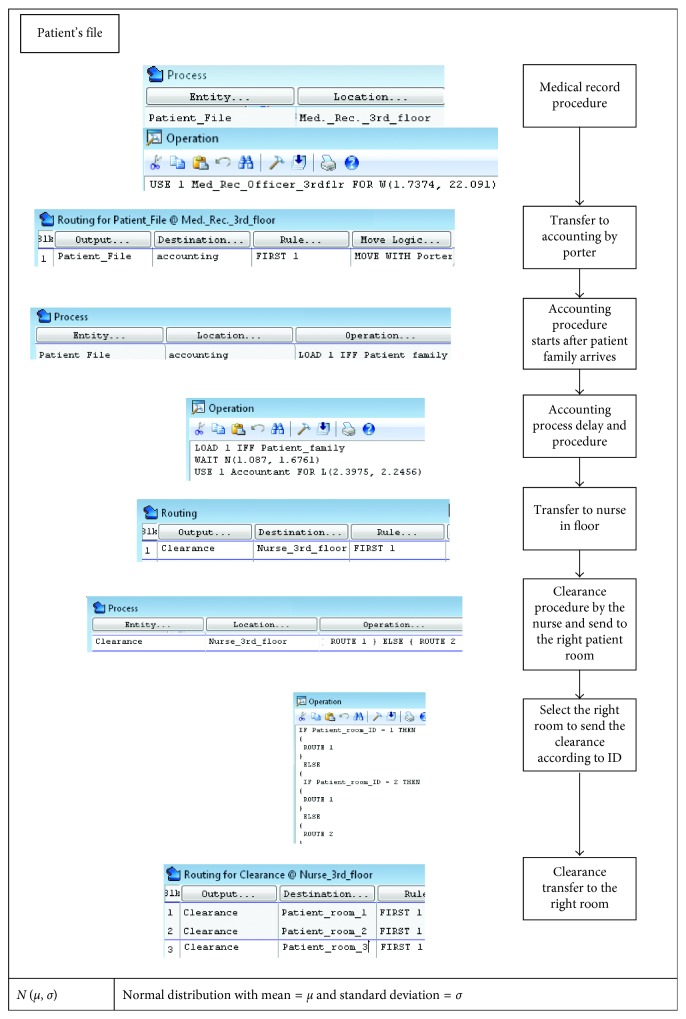
Patient's file tracking.

Detailed code was developed to simulate each step in the discharge process. For illustration, a sample of the simulation code developed is shown in Figure 14. The figure explains the tracking of the patient's file.

In the simulation, the patient's file is represented as an entity called “Patient_file.” The patient's file requires the attention of the medical records officer for *W* (1.7374, 22.091) time, where *W* (*β*, *η*) Weibull distribution with shape parameter = *β* and scale parameter = *η*. This is modeled using “USE 1 Med. Rec. 3rd flr. for *W* (1.7374, 22.091)” for the operation in the location “medical_record_3rd_floor.” The file is then moved to the accounting department. The flow in the accounting is done by the patient's family. This is represented by the load statement “load if patient family.” In this case, the patient file does not receive any attention in the accounting department until the patient's family arrives. Then the accounting process takes *N* (1.1087, 1.43610) in delay time and *L* (2.39750, 2.24560) in attention from the accountant, where *N* (*μ*, *σ*) is normal distribution with mean = *μ* and standard deviation = *σ*, and *L* (*μ*, *σ*) is the lognormal distribution with mean = *μ* and standard deviation = *σ*.

The process results in the clearance entity in the accounting department, moving the patient's file to the third floor in the routing. The clearance is then sent to the specified patient room, according the attribute “Patient_room_ID.”

#### 4.4.2. Verification and Validation of the Model

**Figure 15:**
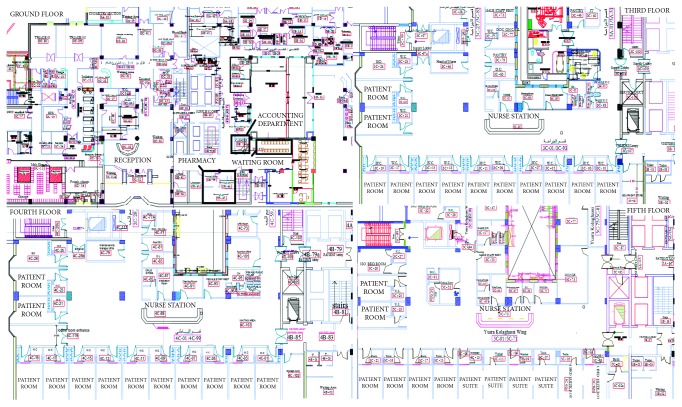
View of the simulation model used for verification.

Verification was initially performed by visualizing the animation of the simulation model flow, entity by entity. All stakeholders' (PF, doctors, nurses, and porters) movement in the hospital was checked to verify the correctness of the simulation model.  Figure 15 shows a snapshot of the simulation model while running in the different floors.

The simulation model was then validated by comparing the output of the model to the actual discharge process in the hospital. We looked at both the number of discharges and the average duration of the discharge process.

**Figure 16:**
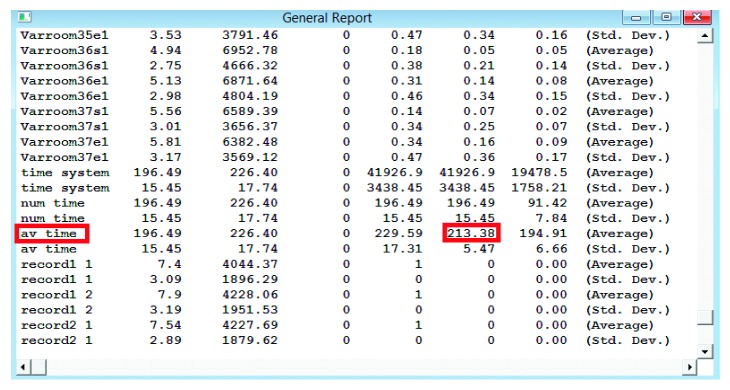
General report from ProModel showing the average time for 100 replications.

The model was run for a simulated period of time equal to one month. Because of the stochastic behavior of the system, a single run would be insufficient to draw an actual estimate from simulation model. Instead, 100 replications were performed, and the average of these runs was evaluated. The simulated average time for the 100 replicates of the discharge process was 213.38 minutes, with standard deviation of 5.47 minutes as shown in Figure 16. The results for one replication were an average time of 213.48 minutes and standard deviation of 76 minutes. The average discharge time observed in the collected data was 215.7 minutes. The error between the real collected data and data from the simulation model is calculated using the following formula:

(2) $$\begin{array}{c} \text{error}=\frac{215.7-213.38}{215.7}=1.08\%. \end{array}$$

#### 4.4.3. Proposed Changes and Time Improvements

After the simulation model was verified and validated, we identified several activities that contributed substantially to increasing the duration of the discharge process. These activities included medication preparation in the pharmacy, waiting for the porter, and the preparation of supplies and equipment. In addition, activities related to physicians, such as late rounds and the fact that physicians do not write prescriptions for medication at the same time as the discharge order, were also important factors adding to the duration of the discharge process.

**Figure 17:**
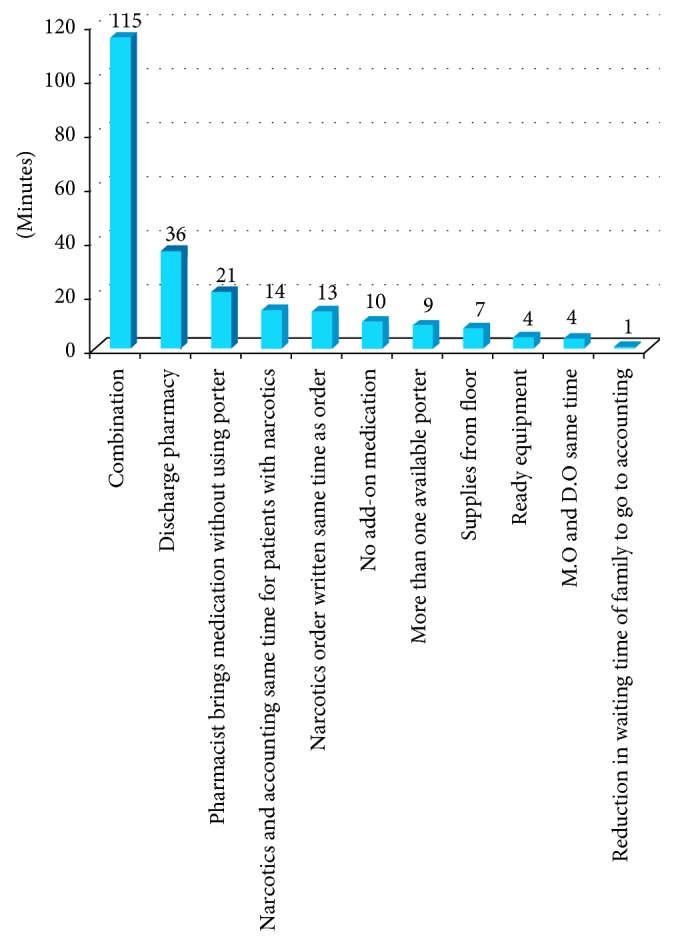
Reduction in time per activity.

Before approaching the hospital management with recommendations for improvements, the simulation model was run to study how improving each activity would affect the discharge process time. The improvements are visualized in a Pareto chart (Figure 17).

The first improvement noted is creating a discharge “fast track” in the pharmacy. This means that the pharmacy would take 30 minutes for medication preparation instead of an average of 88 minutes. In the Pareto chart, it can be seen that this improvement would reduce the total discharge time by an average of about 36 minutes.

Because the pharmacy also waits for the porter's arrival to transport the medication to the patient, even when the medication is ready, we suggested making another porter available, which would save 8.53 minutes. Another suggested solution was eliminating both waiting at the pharmacy and the high variation in porter transportation by assigning the role of transporting the medication to the clinical pharmacist, thus eliminating the nurse's phone call and waiting during delays in the pharmacist's arrival; this would result in a 21-minute reduction, on average, in the total discharge duration.

Announcing the need for equipment one day earlier would lead to an average reduction of 4.2 minutes. Additionally, if supplies were brought to the floor instead of requiring the patient to go to the outpatient clinic, this would save 7.36 minutes.

**Figure 18:**
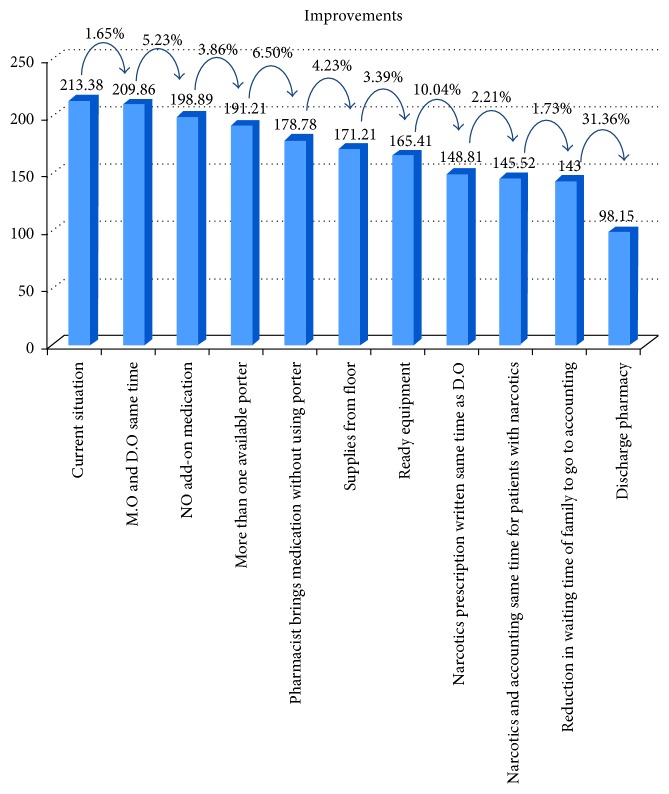
Percentage improvements with the addition of each improvement.

When improvements were applied to all of the activities, the discharge process decreased by about 115 minutes, resulting in a discharge process of about 98 minutes as shown in Figure 18 and a total reduction of approximately 54%.

**Figure 19:**
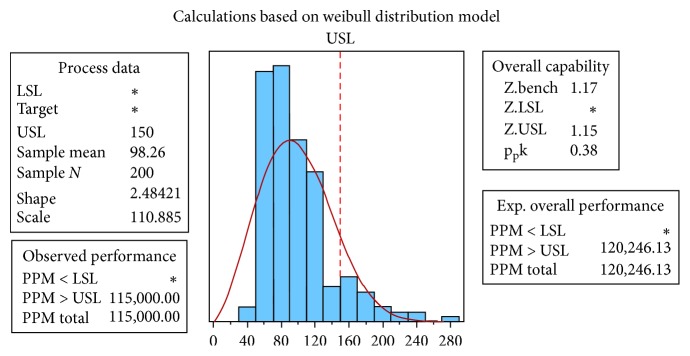
Process capability report after improvement.

A process capability analysis was also performed after improvement, as shown in Figure 19. The *Z*_bench_ is equal to 1.17, which is equivalent to an SQL of 2.67. The SQL value increased from 0.72 to 2.67, meaning that there was a decrease in the number of patients waiting longer than 150 minutes.

### 4.5. “Control” Phase

The last phase of the DMAIC is the “Control” phase. A control plan was put in place to ensure that the improvements would continue in the future. The goals here were to ensure that the processes continue to work well, produce the desired output results, and maintain quality levels.

All organizations experience resistance to change. As LSS are by definition about changing how people work, many LSS efforts are met with resistance. Furthermore, Yih [25] has argued that this resistance is often viewed as insurmountable in healthcare organizations. This gloomy view arises because physicians—one of the most important and most highly constrained resources in hospitals—are mostly autonomous in the management structure and thus immune to incentives typically available in other organizations.

Because of the autonomous nature of their profession, it is difficult for physicians to accept standardization, especially when it goes against their own interests [41]. Physicians do not feel comfortable adopting a standardization initiative unless there is transparent evidence of its impact on patient outcomes [42].

To alleviate physicians' resistance, we performed a stakeholder analysis.

As shown in Table 2, physicians have high levels of influence and impact on the control process steps, but their interest in these issues is low. We recommend involving physicians in the analysis and development of solutions, whether through participation in the improvement team or workshops and meetings presenting and discussing quality improvement issues.

Other stakeholders such as pharmacy workers, the accounting department, and medical records staff members should collaborate in the proposed methods to sustain and control the improvements, for example, using an electronic discharge system. These stakeholders have low-to-moderate interest in leading or initiating any change process and medium impact on the process control and improvement. Patients' family members who usually participate in the discharge process are contacted by the discharge planning coordinator in a one on one meeting, their role in executing an effective discharge process is explained, and any special arrangements are discussed and taken care of.

Change is always unsettling, even when all the parties involved are committed to the outcome. Taking the time to brief the stakeholders ensures cooperation and reduces stress during and immediately following the improvement event [43]. A responsible, accountable, consulted, and informed matrix was utilized to improve communication, convey information about responsibilities, and identify any gaps or redundancies associated with stakeholders' responsibilities, as shown in Table 3.

If all of the stakeholders understand this matrix and take the proper actions accordingly, communication between these people will improve, reducing the waste of time and thus benefiting the patient.

**Figure 20:**
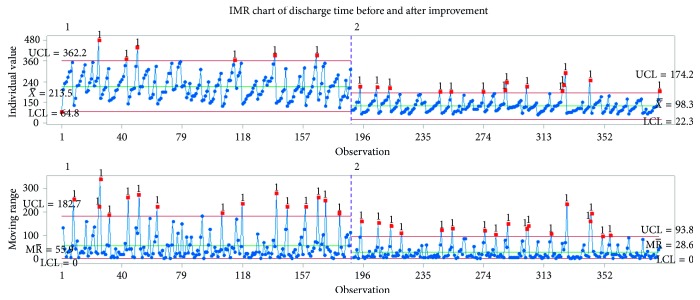
Early indications of success: IMR chart of discharge time before and after improvement.

Control charts were used to monitor the ongoing performance of the key variables. After implementing all of the improvements in the process, a decrease in the mean duration of the discharge process was observed, as seen in Figure 20, which shows the control chart before and after improvement. The mean discharge time decreased by approximately 54%, from 216 minutes to 98 minutes. The lower and upper control limits of the individual values and the moving range also showed a reduction, indicating a more stable process.

A control plan was put in place to ensure that these improvements would continue in the future. Control charts are used to verify compliance.

## 5. Conclusions

6*σ*, combined with the power of DES, has been effectively applied to the improvement of patient discharge processes, an intricate healthcare operational process involving multiple stakeholders. Application of the 6*σ-*DMAIC methodology provided a structured framework to define the project goals, understand the current state, analyze the data to identify the root causes, assess statistically significant improvements, and implement a control plan to maintain improvements in the discharge process.

The patient discharge process was complex and unstandardized and involved multi-department processing and sequential operations. Discharged patients were classified into two groups (standard and complex patients) since their discharge times differed significantly. This was due to the fact that complex patients required extra needs (medical supplies and equipment) and thus extra processing steps and time, many of which can be preplanned and prepared before the discharge process took place.

Leveraging 6*σ* methodology with DES enabled us to get the most out of process improvement initiative. Building a simulation model with the smallest details of the discharge process provided a realistic ideation tool for stimulating and eliciting more solution ideas for consideration. After verification and validation, analysis of the simulation results provided a means for doing scenario comparisons and identifying key process factors affecting performance of the discharge process. With DES, we were able to quantify the levels of improvement that can be anticipated from the different proposed solutions, and thus, offering the KHCC management a variety of solutions. The total reduction in discharge time was approximately 54%.

This project has been extremely challenging, due mainly to the large scope and the complexity of the processes, and the involvement of stakeholders from a variety of levels and across different functional areas. However, understanding process dynamics and improving communication and collaboration between stakeholders based on stakeholder analysis ensures a significant and sustainable impact on operations.

## Figures and Tables

**Table 1:** Summary of tools used in existing work applying Six Sigma to hospitals' discharge process.

Tools used	Case studies			
	Allen et al. [26]	Udayai and Kumar [8]	El-Banna [28]	Vijay [29]
Process maps	1		1	1
Time study		1		1
Brainstorming				1
Checklist		1		1
*Control charts*				
Individual moving range (IMR)	1		1	
*Root cause analysis*				
Cause-and-effect (C & E) matrix	1			
C & E diagram		1	1	
5 why?				1
Brainstorming	1			
Pareto			1	
Simulation			1	
Sigma quality level (SQL)			1	
Design of experiment (DOE)			1	

**Table 2:** Barriers and proposed engagement methods for stakeholders in the discharge process.

Stakeholder	Knowledge of stakeholder regarding the initiative	Interest in the issue (willingness to initiate or lead)	Influence/power (low, medium, high)	Level of engagement	Impact of issue on actor (low, medium, high)	Proposed engagement method
Physician	Medium	Low	High	Involve	High	Increase ownership/workshops/meetings
Nurse	Low	Medium	Medium	Involve	Medium	Workshops/meetings
Patient/patient's family	Medium	High	Low	Involve	High	One on one meetings
Pharmacy	Low	Low	High	Involve	Medium	Electronic system
Accounting department	Low	Low	Medium	Collaborate	Medium	Electronic system
Medical records	Medium	Medium	Medium	Collaborate	Medium	Electronic system

**Table 3:** RACI matrix for stakeholders in the discharge process.

Task	Physician	Nurse	Pharmacy	Accounting department	Suppliers	Medical records	Patient's family
Discharge orders	R/A	I	I			I	I
Medication orders	R/A	I	C/I			I	I
Supplies and equipment orders	A	A	C/I		I	I	I
Prescription preparation	I		R/A	I		I	I
Suppliers preparation	I			I	R/A	I	I
Medical records update	I		I			R/A	I
Bill preparation			I	R/A	I	I	I
Bill settlement			I	I	I	I	R/A

R = responsible; A = accountable; C = consulted; I = informed.
